# Supplementary material for: Genetic liability to psoriasis predicts severe disease outcomes
Source: Genome Med. 2025 Dec 17;18:14. doi: 10.1186/s13073-025-01561-2 (PMC12857016; doi:10.1186/s13073-025-01561-2)
Supplement: Supplementary file 2 — Additional file 2: Supplementary Figures S1-S9 and Supplementary Methods. [file 13073_2025_1561_MOESM2_ESM.docx]

**Supplementary Figures**


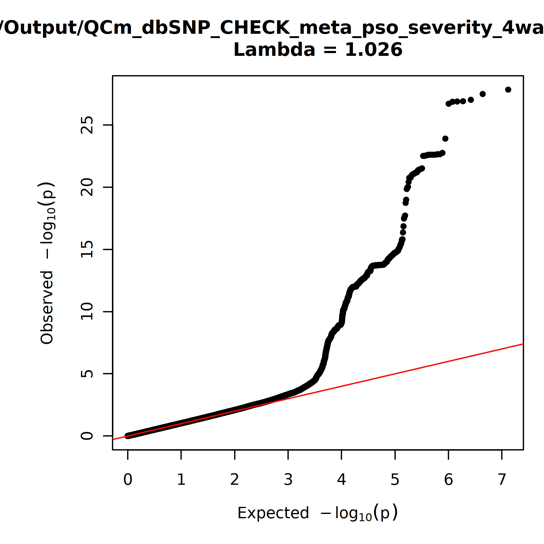


**Figure S1**: Quantile-quantile plot for severe psoriasis GWAS meta-analysis of the four population-based cohorts.


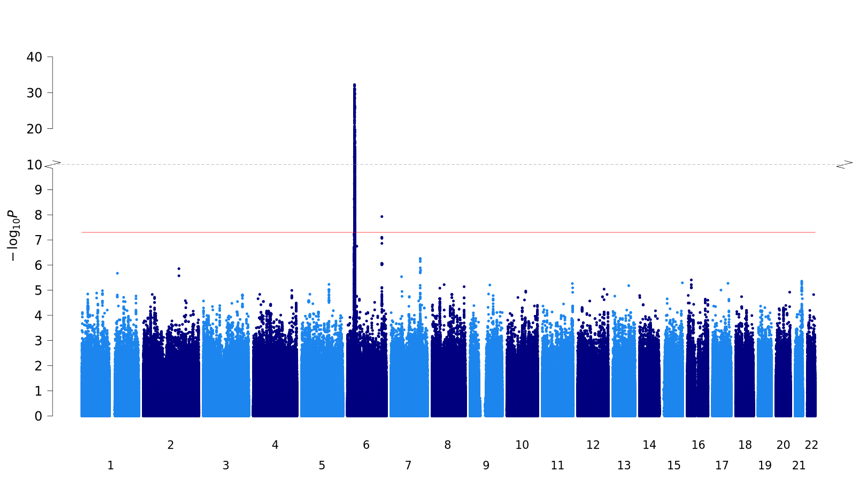

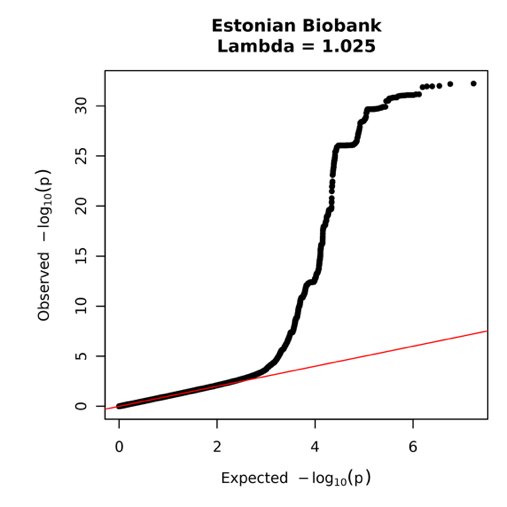

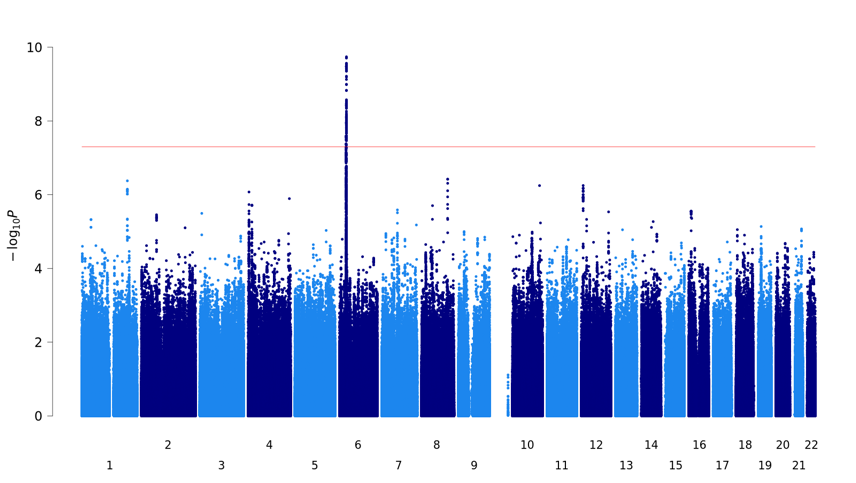

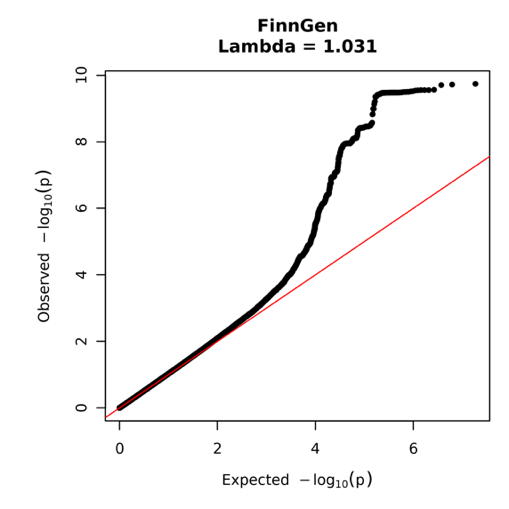

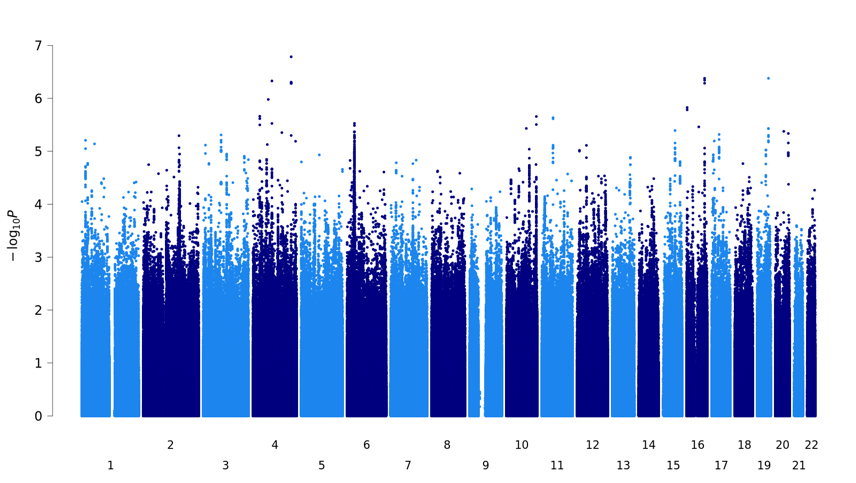

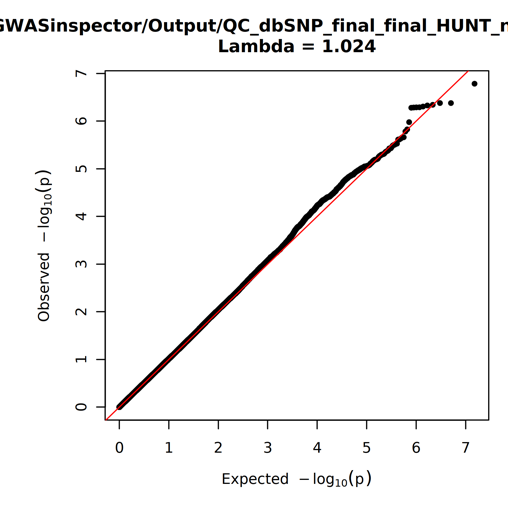

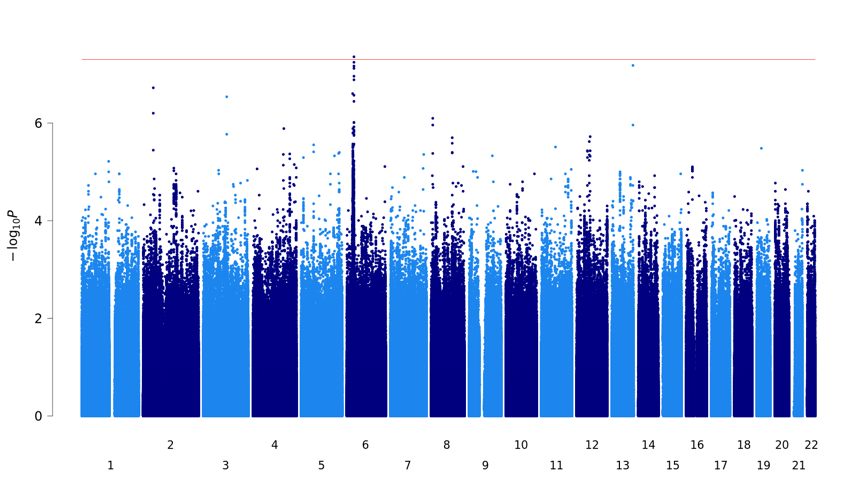

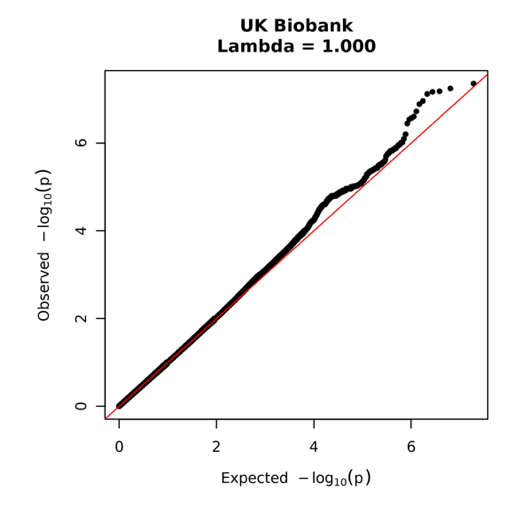


A

B

C

D

**Figure S2**: Manhattan and quantile-quantile plots for severe psoriasis GWAS of four population-based cohorts. Red line on Manhattan plots: – log(P) = 5 × 10^-8^; Red line on QQ plots: x=y; A: Estonian Biobank; B: FinnGen; C: HUNT; D: UK Biobank.


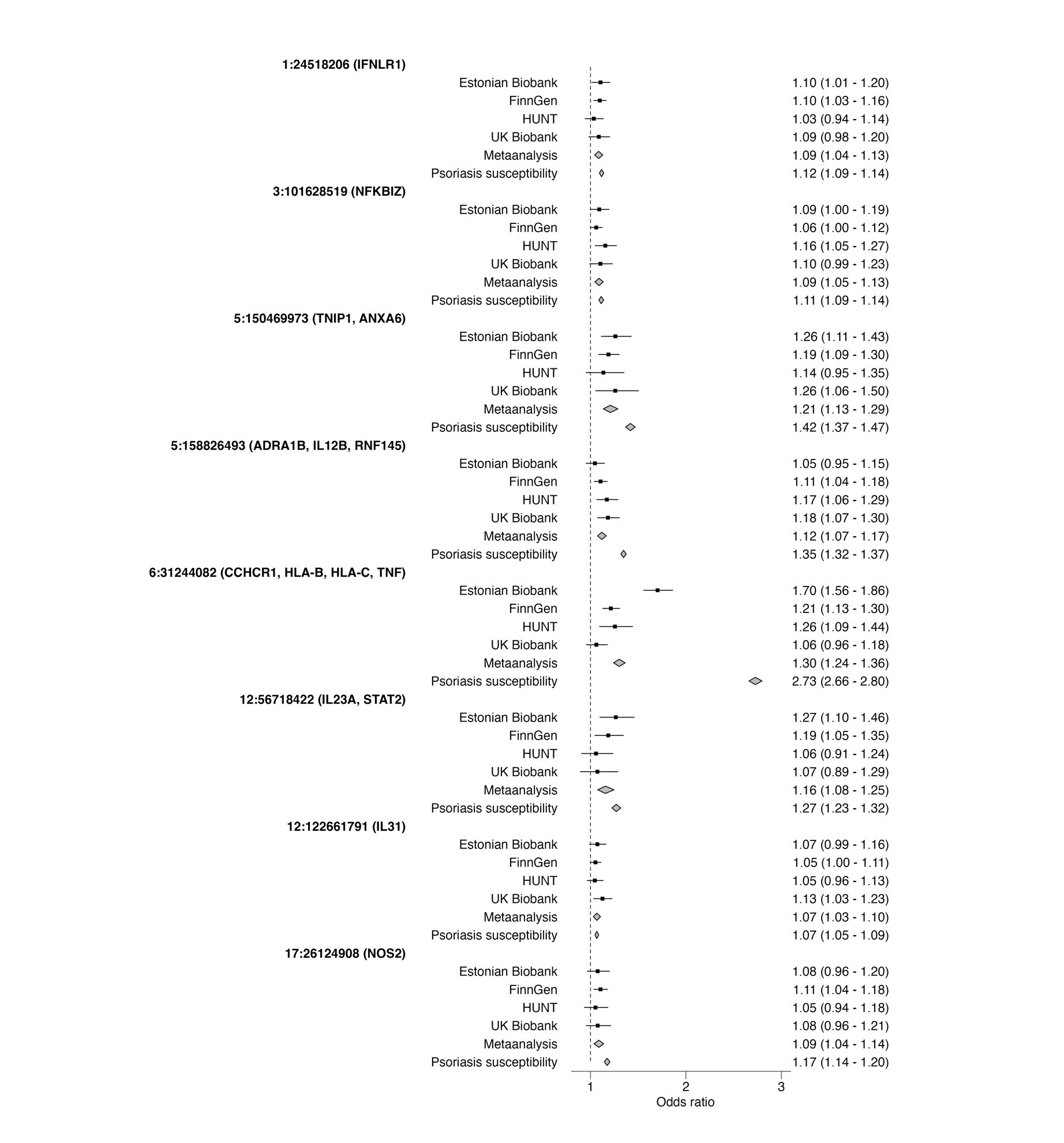


**Figure S3**: Effect size estimates for eight psoriasis susceptibility SNPs significantly associated with severe disease after multiple testing (P<0.0005). Candidate genes are shown in brackets (Dand et al. 2025). Additive per-allele effect sizes presented numerically as: odds ratio (95% confidence interval).


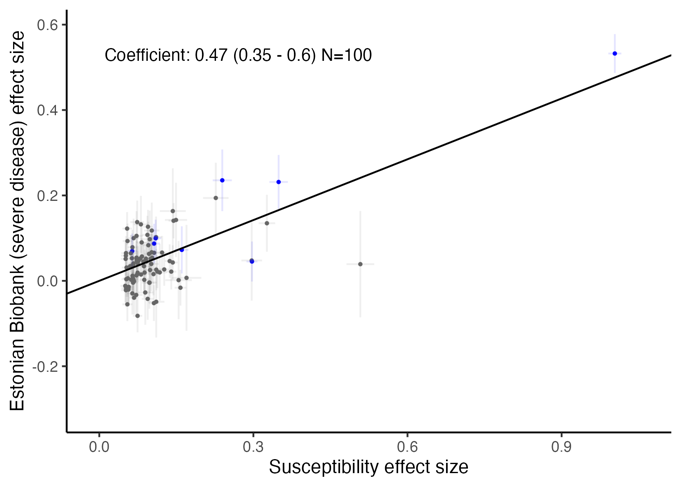

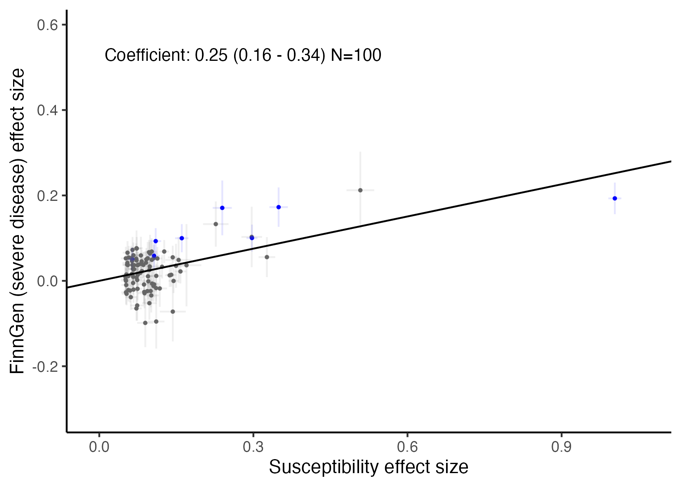

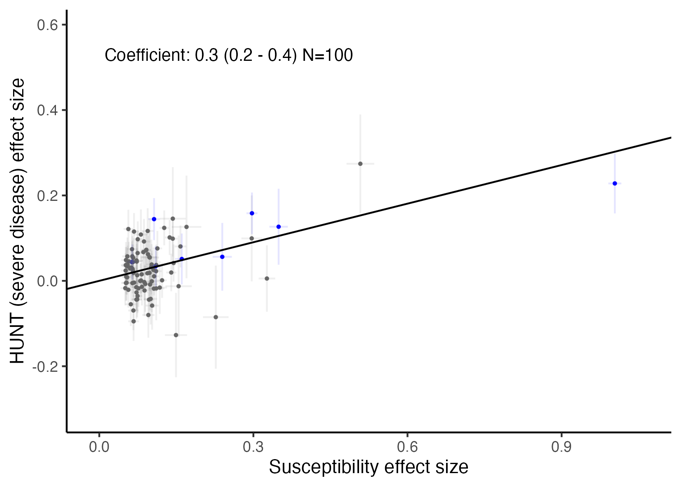

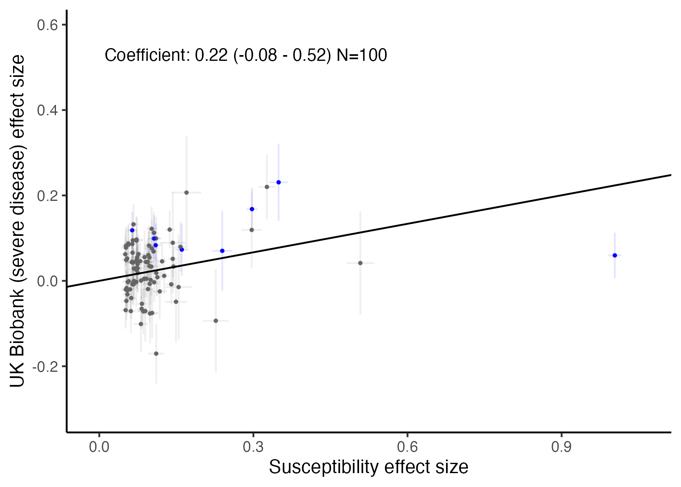


**Figure S4**: Deming regression of severe psoriasis GWAS SNP effect sizes (betas) against psoriasis susceptibility effect sizes (betas), across four different population-based cohorts. SNPs with meta-analysis Bonferroni-corrected P-values < 0.05 are coloured blue.


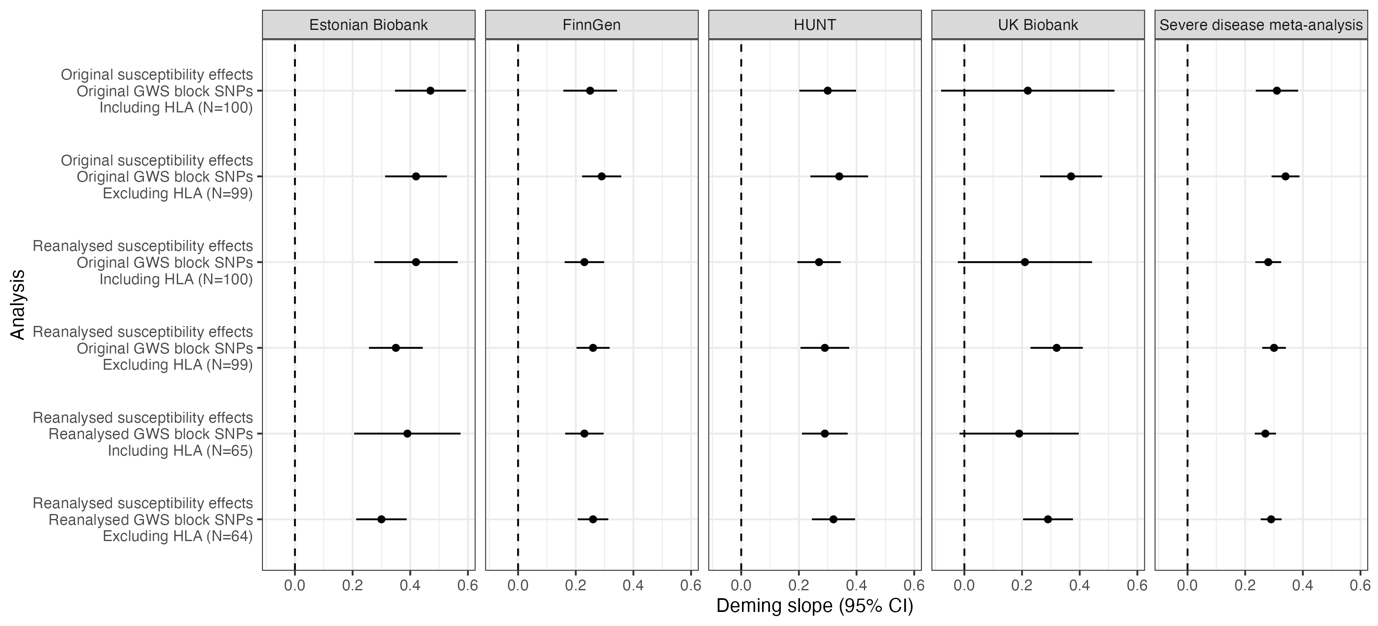


**Figure S5**: Deming regression slopes comparing effect sizes of psoriasis susceptibility SNPs to effect sizes in severe disease for the meta-analysis and all four population-based cohorts individually. Sensitivity analyses where SNP selection and weighting have been adjusted to reflect complete independence from the population-based datasets are shown, as well as replication excluding the lead MHC SNP.


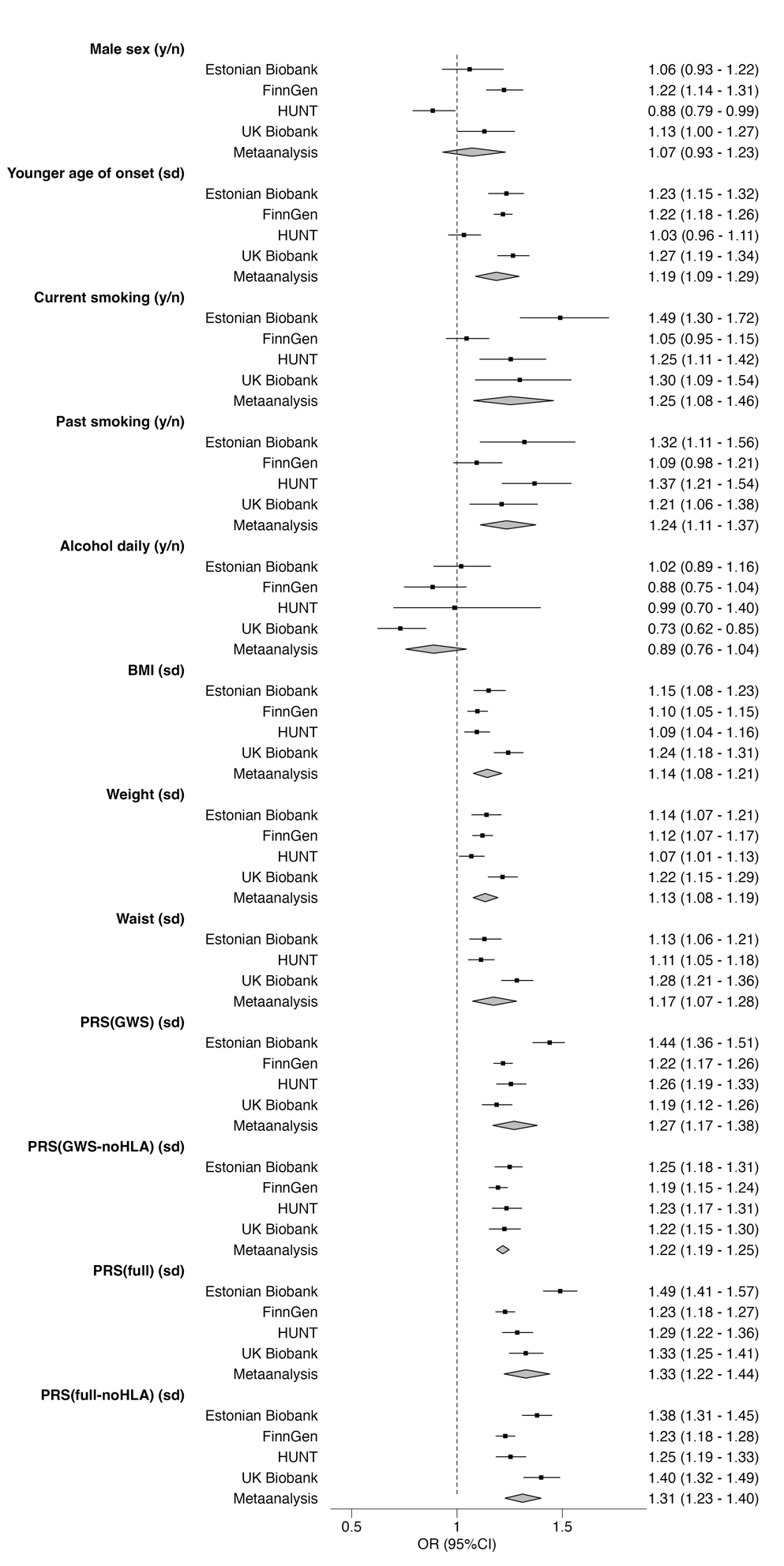


**Figure S6:** Cohort-specific comparison of marginal effects of non-genetic factors and PRS_full_ and PRS_GWS_ with severe psoriasis. Effect estimates are derived from a meta-analysis of unadjusted logistic regression models comparing severe to non-severe psoriasis. y/n: effect size estimated for presence of exposure (“yes”) relative to absence (”no”); sd: effect size estimated per standard deviation change in continuous exposure within the psoriasis population; OR: Odds ratio. Effect sizes presented numerically as odds ratio (with 95% confidence intervals). Standard deviations for each quantitative factor in each cohort are detailed in **Additional file 1: Table S10**.


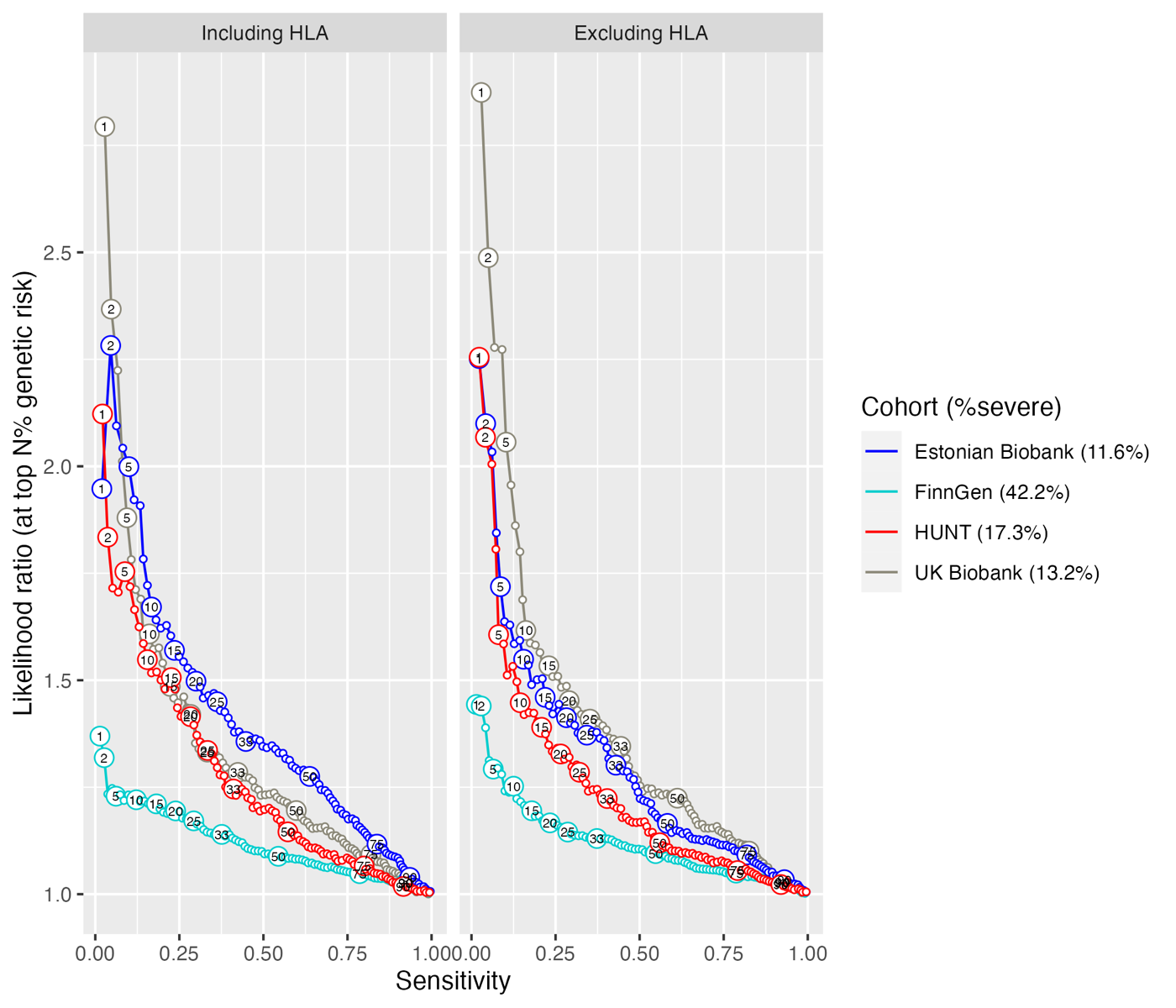


**Figure S7**: Likelihood ratio plot against sensitivity, using top *N*% of psoriasis susceptibility PRS_full_ as a “diagnostic test” in each of the European biobank cohorts. Top *N* percentage indicated as labels on selected points on the plot; *Sensitivity* = [number of severe individuals captured at PRS threshold] / [total severe individuals in cohort]; *Likelihood ratio* = [proportion of individuals captured by PRS who are severe] / [baseline rate of severe disease in cohort]


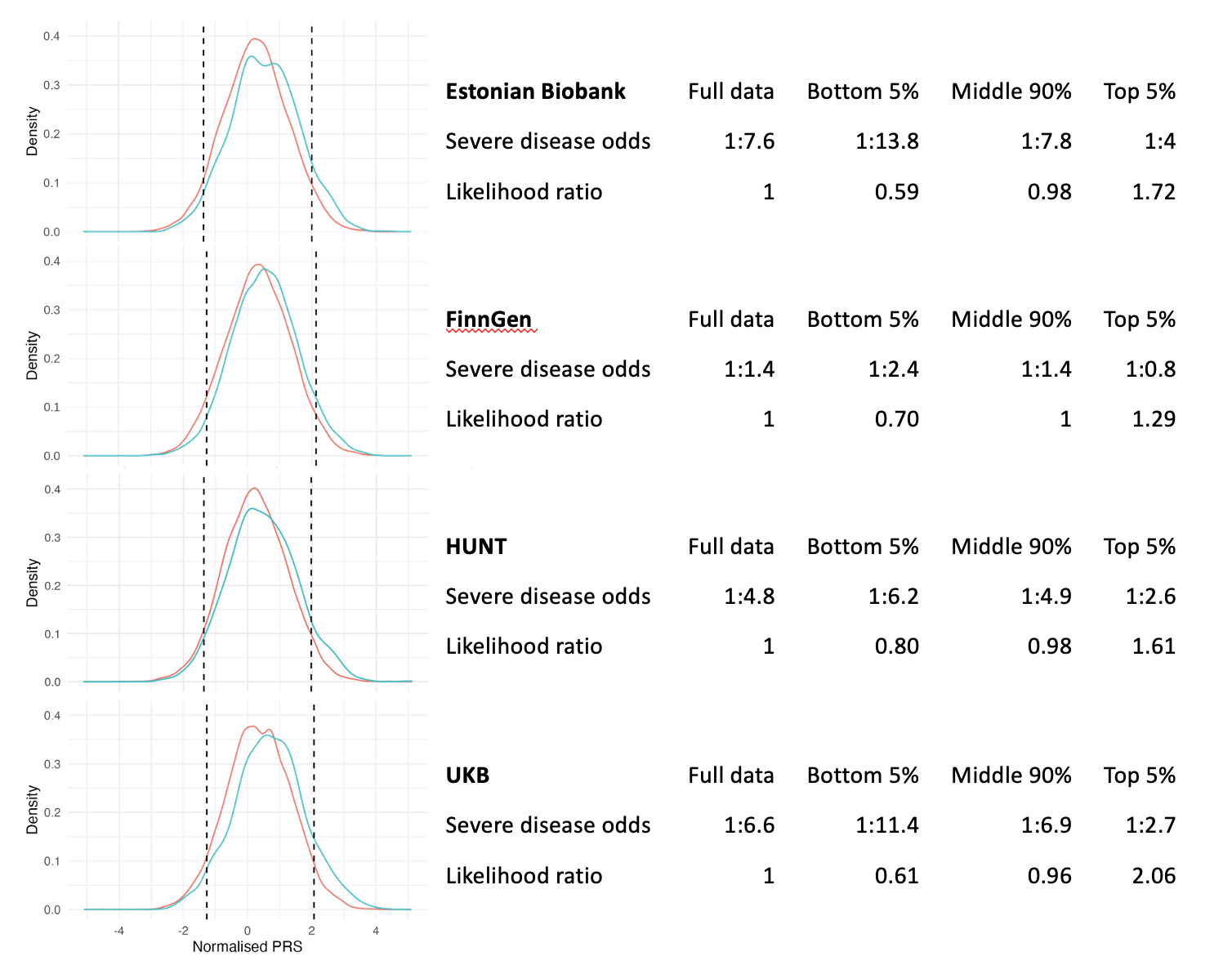


**Figure S8**: Sensitivity analysis showing distributions and performance of susceptibility PRS_full-noHLA_ in predicting severe psoriasis cases within psoriasis population in each biobank study. Cut-offs (dashed lines) displayed for individuals within the top 5%, middle 90% and bottom 5% of the (within-dataset) PRS distribution. Red line indicates PRS distribution of non-severe psoriasis population. Blue line indicates PRS distribution of severe psoriasis population. *Severe disease odds*: ratio of individuals with severe disease to individuals without severe disease. *Likelihood ratio*: ratio between the severe disease odds in each PRS group and the severe disease odds for all psoriasis cases.


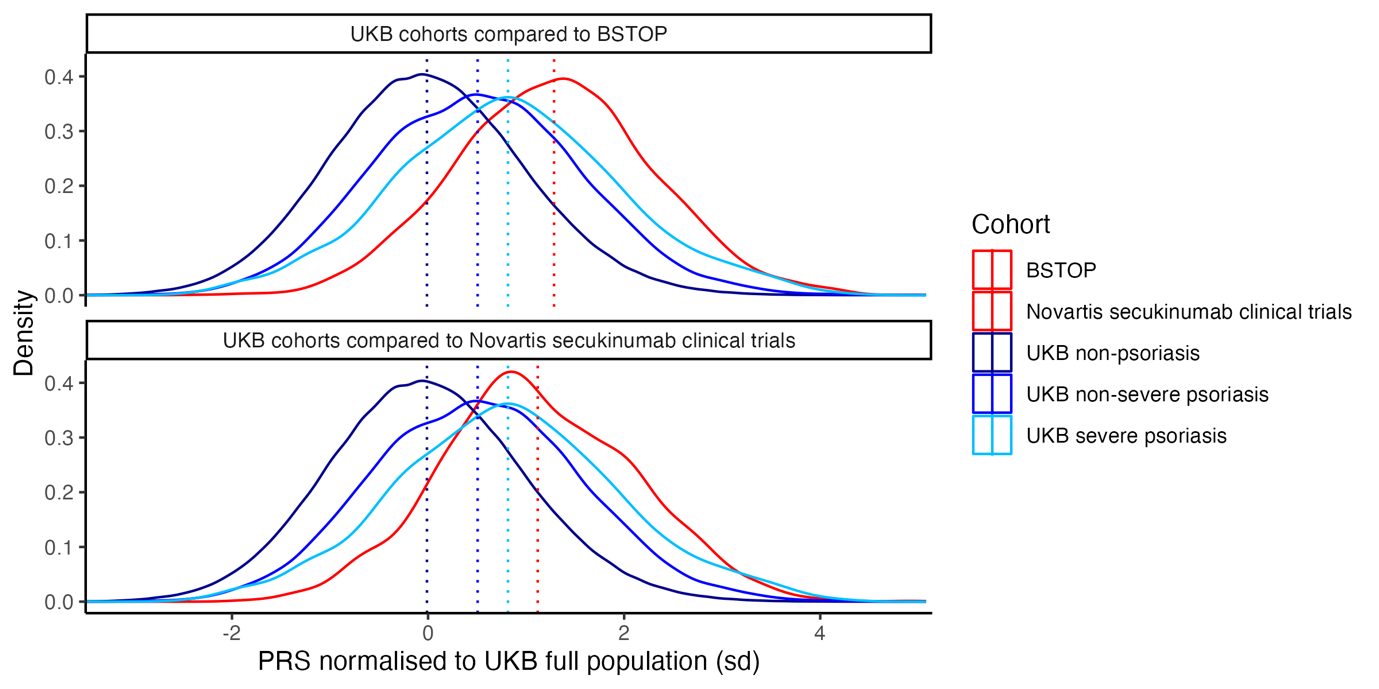


**Figure S9**: Comparison of PRS*_full_ distributions between UK Biobank populations (N_non-psoriasis_ = 449 868; N_non-severe psoriasis_ = 8 183; N_severe psoriasis_ = 1 243) and external validation cohorts (BSTOP registry [N = 4 151] and Novartis secukinumab clinical trials [N = 1 461]).

**Supplementary Methods**

Genotyping and GWAS

*Estonian Biobank*

The EstBB samples were genotyped at the Core Genotyping Lab of the Institute of Genomics, University of Tartu, using Illumina global screening arrays v1.0, v2.0 and v2.0_EST. Individuals with <95% call rate or sex mismatch between genetic sex and sex recorded in phenotype data were excluded from the analysis. Before imputation, variants were filtered by call rate < 95%, Hardy–Weinberg equilibrium P < 1 × 10-4 (autosomal variants only), and minor allele frequency < 1%. Prephasing was performed using Eagle v2.4.1 software, and imputation was performed using Beagle v.5.4 with the use of an Estonian population–specific imputation reference panel built from 2,297 whole-genome sequencing samples. Association testing was performed with a mixed-effects logistic regression model (REGENIE v3.0.3), including age, sex and ten principal components as covariates.

*FinnGen*

FinnGen (www.finngen.fi) is public-private research project that combines genomic data with Finnish health registry data. The project aims to genotype 500,000 Finnish individuals. FinnGen is a partnership of Finnish biobanks, their background organizations, pharmaceutical industry partners, and Finnish biobank cooperative (FINBB) (https://www.finngen.fi/en/partners). Data Freeze release 10, used in this analysis, contains 412,181 individuals with genotype and phenotype information.

Genotyping and quality control in FinnGen is described at <https://finngen.gitbook.io/documentation/methods/genotype-imputation/genotype-data>. Briefly, samples were genotyping using Illumina (Illumina Inc., San Diego, CA, USA) and Affymetrix arrays (Thermo Fisher Scientific, Santa Clara, CA, USA). Samples were excluded based on the following criteria: no sex information or ambiguous sex, duplicates, >2% variant missingness, excess heterozygosity in common variants (MAF > 5%; +-3 standard deviation per batch). Samples with excess relatedness ((π ) ̂>0.1) were also excluded in two rounds, first removing samples with > 500 related samples, then removing those remaining with > 50 counts. Variants with >2% missingness or Hardy-Weinberg equilibrium (HWE) P-value< 1x10^-6^ within a batch were removed. Variants were removed from all batches based on the following criteria: HWE P-value< 1x10^-10^ across all batches, missingness > 3% in any batch, or more than 15% of batches with missingness > 4%. Genotypes were pre-phased using Eagle 2.3.5 with the number of conditioning haplotypes set to 20,000. Genotype were subsequently imputed using the SISu v.4.0 reference panel using Beagle 4.1 (version 08Jun17.d8b). SISu 4.0 is a Finnish reference panel consisting of 8,554 whole genome sequences.

GWAS were performed using logistic mixed model implemented in REGENIE (v. 2.2.4) in the FinnGen sandbox environment. Both additive and dominant models were run, adjusting for age, sex, top 10 principal components, and genotyping batches. Specific genotyping batches were included as covariate if they were using in at least 10 cases or 10 controls. Variants with a minor allele count >= 5 were included in the analysis.

*HUNT*

The Trøndelag Health Study (HUNT) is a population-based cohort study carried out at four time points over approximately 40 years (HUNT1 [1984-1986], HUNT2 [1995-1997], HUNT3 [2006-2008] and HUNT4 [2017-2019]) (1,2). All inhabitants aged 20 years and over residing in Trøndelag County in Norway were invited to participate.

Participants from HUNT2-4 were genotyped using one of four different Illumina HumanCoreExome arrays (HumanCoreExome12 v1.0, HumanCoreExome12 v1.1, UM HUNT Biobank v1.0 and UM HUNT Biobank v2.0) (3). Genotype calling was performed with GenTrain v.2.0 in GenomeStudio v.2011.1 (Illumina). Samples with <99% genotype calls, with large chromosomal copy number variants, contamination >2.5% as estimated with BAF Regress (4), with genotypic and phenotypic sex discordance, and not of European ancestry were excluded, leaving 87,028 genotyped subjects. Genetic variants out of Hardy-Weinberg equilibrium (p-value <0.0001) were excluded. Samples were phased with Eagle2 v2.0.5 (https://alkesgroup.broadinstitute.org/Eagle/) and imputed with the positional Burrows-Wheeler transform (PBWT) v3.1 (https://github.com/richarddurbin/pbwt) (5).

GWAS was run in SAIGE v1.0.3 (6), using sex, birth year, genotyping batch and 10 ancestry principal components as covariates. Variants with MAF >3.2e-04 were included in the analyses, and dosages were used for imputed variants.

*UK Biobank*

The UK Biobank central team performed genotype calling and imputation. Genotyping was performed using the Affymetrix UK BiLEVE Axiom array (n ∼50,000) and the Affymetrix UK Biobank Axiom array (n ∼450,000) (7). Based on quality control metrics provided by UK Biobank, we removed samples that exhibited sex mismatch, high relatedness (>3^rd^ degree) to a large number (>200) of individuals, heterozygosity or missingness outliers, non-European individuals (according to kmeans cluster analysis) and withdrawals, leaving 462,817 individuals. Genome-wide imputation was performed by the UK Biobank central team using IMPUTE2 software and a reference panel derived from UK10K and 1,000 Genomes phase 3 haplotypes (8,9).

BOLT-LMM (10) was used to conduct genome-wide association testing, controlling for genotyping array type, sex, year of birth and 10 genotyping principal components. Effect sizes were approximated to the logistic scale using the formula: log(OR) = β / (μ * (1 - μ)), where μ = case fraction. Standard errors were also converted by dividing by (μ * (1 - μ)).

*BSTOP*

BSTOP is an ongoing prospective observational study of patients with moderate‒severe plaque psoriasis across >70 UK dermatology centres, which includes biological sample collection. Genome-wide genotyping array data were generated for BSTOP participants at the Institute of Psychiatry, Psychology and Neuroscience Genomics and Biomarker Facility at King’s College London (United Kingdom) using Illumina HumanOmniExpressExome-8, version 1.2, 1.3 and 1.6, and BeadChips (Illumina, San Diego, CA). Basic quality control and batch merging were performed using the process described in detail elsewhere (11,12).

*Novartis secukinumab clinical trials*

Novartis data was collected from five clinical trials of secukinumab in psoriasis (details available on https://clinicaltrials.gov/): CAIN457A2302 (NCT01365455), CAIN457A2303 (NCT01358578), CAIN457A2304 (NCT01406938), CAIN457A2223 (NCT01537432), CAIN457A2403 (NCT03553823). Genotyping was carried out on the Illumina Global Screening array as previously described (13). Basic quality control was performed and samples were imputed using Beagle 5.2 (14) and the 1,000 Genomes reference panel (15). Imputed variants were filtered to dosage R-squared (DR2) >0.5, missingness <0.1, minor allele frequency >1% and subset to biallelic variants. Subsequently, samples were classified into continental ancestry groups by projecting data on principal components of the 1000 Genomes reference dataset and subsequently subset to samples from European descent.

**References**

1. Krokstad S, Langhammer A, Hveem K, Holmen TL, Midthjell K, Stene TR, et al. Cohort profile: The HUNT study, Norway. Int J Epidemiol. 2013;

2. Åsvold BO, Langhammer A, Rehn TA, Kjelvik G, Grøntvedt TV, Sørgjerd EP, et al. Cohort Profile Update: The HUNT Study, Norway. Int J Epidemiol. 2023 Feb 8;52(1):e80–91.

3. Brumpton BM, Graham S, Surakka I, Skogholt AH, Løset M, Fritsche LG, et al. The HUNT study: A population-based cohort for genetic research. Cell Genomics. 2022 Oct;2(10):100193.

4. Jun G, Flickinger M, Hetrick KN, Romm JM, Doheny KF, Abecasis GR, et al. Detecting and Estimating Contamination of Human DNA Samples in Sequencing and Array-Based Genotype Data. The American Journal of Human Genetics. 2012 Nov;91(5):839–48.

5. Durbin R. Efficient haplotype matching and storage using the positional Burrows–Wheeler transform (PBWT). Bioinformatics. 2014 May 1;30(9):1266–72.

6. Zhou W, Nielsen JB, Fritsche LG, Dey R, Gabrielsen ME, Wolford BN, et al. Efficiently controlling for case-control imbalance and sample relatedness in large-scale genetic association studies. Nat Genet. 2018 Sep 13;50(9):1335–41.

7. Bycroft C, Freeman C, Petkova D, Band G, Elliott LT, Sharp K, et al. The UK Biobank resource with deep phenotyping and genomic data. Nature. 2018 Oct 10;562(7726):203–9.

8. Howie BN, Donnelly P, Marchini J. A Flexible and Accurate Genotype Imputation Method for the Next Generation of Genome-Wide Association Studies. PLoS Genet. 2009 Jun 19;5(6):e1000529.

9. Howie B, Marchini J, Stephens M. Genotype Imputation with Thousands of Genomes. G3 Genes|Genomes|Genetics. 2011 Nov 1;1(6):457–70.

10. Loh PR, Tucker G, Bulik-Sullivan BK, Vilhjálmsson BJ, Finucane HK, Salem RM, et al. Efficient Bayesian mixed-model analysis increases association power in large cohorts. Nat Genet. 2015 Mar 2;47(3):284–90.

11. Dand N, Duckworth M, Baudry D, Russell A, Curtis CJ, Lee SH, et al. HLA-C*06:02 genotype is a predictive biomarker of biologic treatment response in psoriasis. Journal of Allergy and Clinical Immunology. 2019 Jun;143(6):2120–30.

12. Dand N, Stuart PE, Bowes J, Ellinghaus D, Nititham J, Saklatvala JR, et al. GWAS meta-analysis of psoriasis identifies new susceptibility alleles impacting disease mechanisms and therapeutic targets. Nat Commun. 2025 Feb 28;16(1):2051.

13. Zhang C, Shestopaloff K, Hollis B, Kwok CH, Hon C, Hartmann N, et al. Response to anti-IL17 therapy in inflammatory disease is not strongly impacted by genetic background. Am J Hum Genet. 2023 Oct 5;110(10):1817–24.

14. Browning BL, Zhou Y, Browning SR. A One-Penny Imputed Genome from Next-Generation Reference Panels. Am J Hum Genet. 2018 Sep 6;103(3):338–48.

15. Byrska-Bishop M, Evani US, Zhao X, Basile AO, Abel HJ, Regier AA, et al. High-coverage whole-genome sequencing of the expanded 1000 Genomes Project cohort including 602 trios. Cell. 2022 Sep 1;185(18):3426-3440.e19.
